# Editorial: Application of machine learning in the diagnosis of dementia, volume II

**DOI:** 10.3389/fneur.2025.1603844

**Published:** 2025-05-23

**Authors:** Atsushi Inoue

**Affiliations:** Care XDX Center, Kyushu Institute of Technology, Kitakyushu, Japan

**Keywords:** machine learning, diagnosis, dementia, prevention, prediction

While the number of patients with dementia is rapidly increasing and becoming a social issue on a global scale, there is currently no effective cure for dementia. Hence the emphasis is on preventing its onset, especially at the early stages. This is the second volume of the special issue that introduces use of cutting-edge information technology such as Artificial Intelligence (AI), Machine Learning (ML) especially the Deep Learning, and sensing technologies such as Electroencephalogram (EEG) for the early diagnosis of dementia and its improvement of accuracy, which hopefully contributes to a decrease with some global impacts the number of the patients with dementia.

Six papers have been accepted in this volume, that makes eleven papers total with five from the previous one. Five of the six use machine learning with the three for detection and prediction purposes. Three of those five use the modern deep learning algorithms while the remaining two use the classic algorithms. One unique approach is with EEG and a simplified version (a shallow architecture) of the deep learning neural networks in order to detect an early stage of dementia. We notice more contributions using AI and ML at this time.

Advancement of AI never stops but rather accelerates further. Since 2020, generative AI, especially Generative Pre-trained Transformer (GPT) has arisen and made significant impacts. That has been changing the entire game of information technologies that centers natural languages for coding and various task performance. We are very excited that the wave of this new AI will reach the diagnosis of dementia soon and even the medicine in general. We believe that AI and medical staff collaboratively perform various medical operations and tasks very near future.

